# How much should we sequence? An analysis of the Swiss SARS-CoV-2 surveillance effort

**DOI:** 10.1128/spectrum.03628-23

**Published:** 2024-03-18

**Authors:** Fanny Wegner, Blanca Cabrera-Gil, Araud Tanguy, Christiane Beckmann, Niko Beerenwinkel, Claire Bertelli, Matteo Carrara, Lorenzo Cerutti, Chaoran Chen, Samuel Cordey, Alexis Dumoulin, Louis du Plessis, Marc Friedli, Yannick Gerth, Gilbert Greub, Adrian Härri, Hans Hirsch, Cedric Howald, Michael Huber, Alexander Imhof, Laurent Kaiser, Verena Kufner, Stephen L. Leib, Karoline Leuzinger, Etleva Lleshi, Gladys Martinetti, Mirjam Mäusezahl, Milo Moraz, Richard Neher, Oliver Nolte, Alban Ramette, Maurice Redondo, Lorenz Risch, Lionel Rohner, Tim Roloff, Pascal Schläepfer, Katrin Schneider, Franziska Singer, Valeria Spina, Tanja Stadler, Erik Studer, Ivan Topolsky, Alexandra Trkola, Daniel Walther, Nadia Wohlwend, Cinzia Zehnder, Aitana Neves, Adrian Egli, the SPSP consortium

**Affiliations:** 1 Institute of Medical Microbiology, University of Zurich, Zurich, Switzerland; 2 Clinical Bioinformatics, SIB Swiss Institute of Bioinformatics, Geneva, Switzerland; 3 Genesupport, Geneva, Switzerland; 4 Viollier AG, Allschwil, Switzerland; 5 Department of Biosystems Science and Engineering, ETH Zurich, Switzerland & SIB Swiss Institute of Bioinformatics, Basel, Switzerland; 6 Clinical Microbiology, University Hospital, Lausanne, Switzerland; 7 NEXUS Personalized Health Technologies, ETH Zurich, Switzerland & SIB Swiss Institute of Bioinformatics, Basel, Switzerland; 8 Health2030 Genome Center, Geneva, Switzerland; 9 Laboratory of Virology, Geneva University Hospitals, Geneva, Switzerland; 10 Valais Hospital, Central Institute, Sion, Switzerland; 11 Humanmedizinische Mikrobiologie, Zentrum für Labormedizin, St. Gallen, Switzerland; 12 Biolytix, Witterswil, Switzerland; 13 Clinical Virology, University Hospital, Basel, Switzerland; 14 Institute of Medical Virology, University of Zurich, Zurich, Switzerland; 15 Spitalregion Oberaargau, Langenthal, Switzerland; 16 Institute for Infectious Diseases (IFIK), University of Bern, Bern, Switzerland; 17 Microbiology Department, Synlab, Bioggio, Switzerland; 18 Department of Laboratory Medicine, Ente Ospedaliero Cantonale, Bellinzona, Switzerland; 19 Federal Office of Public Health, Bern, Switzerland; 20 Biozentrum, University of Basel, Switzerland & SIB Swiss Institute of Bioinformatics, Basel, Switzerland; 21 Labor Dr. Risch, Buchs, Switzerland; 22 Clinical Microbiology, University Hospital, Basel, Switzerland; Foundation for Innovative New Diagnostics, Geneve, Switzerland

**Keywords:** SARS-CoV-2, surveillance studies, genomic surveillance, whole genome sequencing, modeling, molecular epidemiology, public health, platform

## Abstract

During the SARS-CoV-2 pandemic, many countries directed substantial resources toward genomic surveillance to detect and track viral variants. There is a debate over how much sequencing effort is necessary in national surveillance programs for SARS-CoV-2 and future pandemic threats. We aimed to investigate the effect of reduced sequencing on surveillance outcomes in a large genomic data set from Switzerland, comprising more than 143k sequences. We employed a uniform downsampling strategy using 100 iterations each to investigate the effects of fewer available sequences on the surveillance outcomes: (i) first detection of variants of concern (VOCs), (ii) speed of introduction of VOCs, (iii) diversity of lineages, (iv) first cluster detection of VOCs, (v) density of active clusters, and (vi) geographic spread of clusters. The impact of downsampling on VOC detection is disparate for the three VOC lineages, but many outcomes including introduction and cluster detection could be recapitulated even with only 35% of the original sequencing effort. The effect on the observed speed of introduction and first detection of clusters was more sensitive to reduced sequencing effort for some VOCs, in particular Omicron and Delta, respectively. A genomic surveillance program needs a balance between societal benefits and costs. While the overall national dynamics of the pandemic could be recapitulated by a reduced sequencing effort, the effect is strongly lineage-dependent—something that is unknown at the time of sequencing—and comes at the cost of accuracy, in particular for tracking the emergence of potential VOCs.

**IMPORTANCE:**

Switzerland had one of the most comprehensive genomic surveillance systems during the COVID-19 pandemic. Such programs need to strike a balance between societal benefits and program costs. Our study aims to answer the question: How would surveillance outcomes have changed had we sequenced less? We find that some outcomes but also certain viral lineages are more affected than others by sequencing less. However, sequencing to around a third of the original effort still captured many important outcomes for the variants of concern such as their first detection but affected more strongly other measures like the detection of first transmission clusters for some lineages. Our work highlights the importance of setting predefined targets for a national genomic surveillance program based on which sequencing effort should be determined. Additionally, the use of a centralized surveillance platform facilitates aggregating data on a national level for rapid public health responses as well as post-analyses.

## INTRODUCTION

The emergence of SARS-CoV-2 rapidly became one of the most challenging public health situations for national health agencies around the world. Initially, diagnostic capacities were limited and had to be established. As it became clear that the pandemic was not going to be contained quickly, the evolution of the virus became an important focus of attention as new variants with different worrisome properties emerged, such as higher transmission rates or reduced vaccine effectiveness. Diagnostic assays had to be adapted to distinguish these new emerging viral variants. Classic epidemiology based on incidence data does not provide sufficient resolution and information to direct public health policies in the face of an ever-changing virus (1). Genome sequencing, meanwhile, can help guide responses on multiple fronts: it allows the analysis of pathogen evolution and transmission and the detection of outbreaks, as well as informs the development and optimization of diagnostic assays and vaccines—all of which in turn inform public health interventions (1–11).

Consequently, significant effort was directed toward capacity building and creating a fast and reliable surveillance system that allowed the tracking and tracing of (novel) SARS-CoV-2 variants. In Switzerland, individual laboratories quickly stepped up to the challenge of genomic surveillance, but without official targets. This changed in March 2021 with the establishment of the national genomic surveillance program by the Federal Office of Public Health (FOPH), which aimed to sequence 2,000–3,000 samples per week. Due to changed circumstances such as the wide availability of vaccines, this target was adjusted in April 2022 to 500 samples per week with a focus on hospitalized patients (Fig. S1). To date, Switzerland has shared almost 168k genome sequences with GISAID (https://gisaid.org/submission-tracker-global/, last accessed on 10 February 2024), making it the eighth country in the world by number of submitted sequences when normalized by population. Nationally, the Swiss Pathogen Surveillance Platform (SPSP) has become the officially mandated Swiss SARS-CoV-2 Data Hub by the FOPH, both providing a national platform and sharing data with other databases such as GISAID and ENA (12, 13).

The number of sequences influences how early emerging viral lineages can be detected (14). Using genomic data from SPSP collected between the onset of the pandemic in February 2020 and the beginning of August 2022, we aimed to describe the national surveillance effort and explore how the amount of sequencing affects key surveillance outcomes: (i) first detection of variants of concern (VOCs), (ii) speed of introduction of VOCs, (iii) diversity of lineages, (iv) first cluster detection of VOCs, (v) density of active clusters, and (vi) geographic spread of clusters.

## RESULTS

### Genomic surveillance in Switzerland

**Fig 1:**
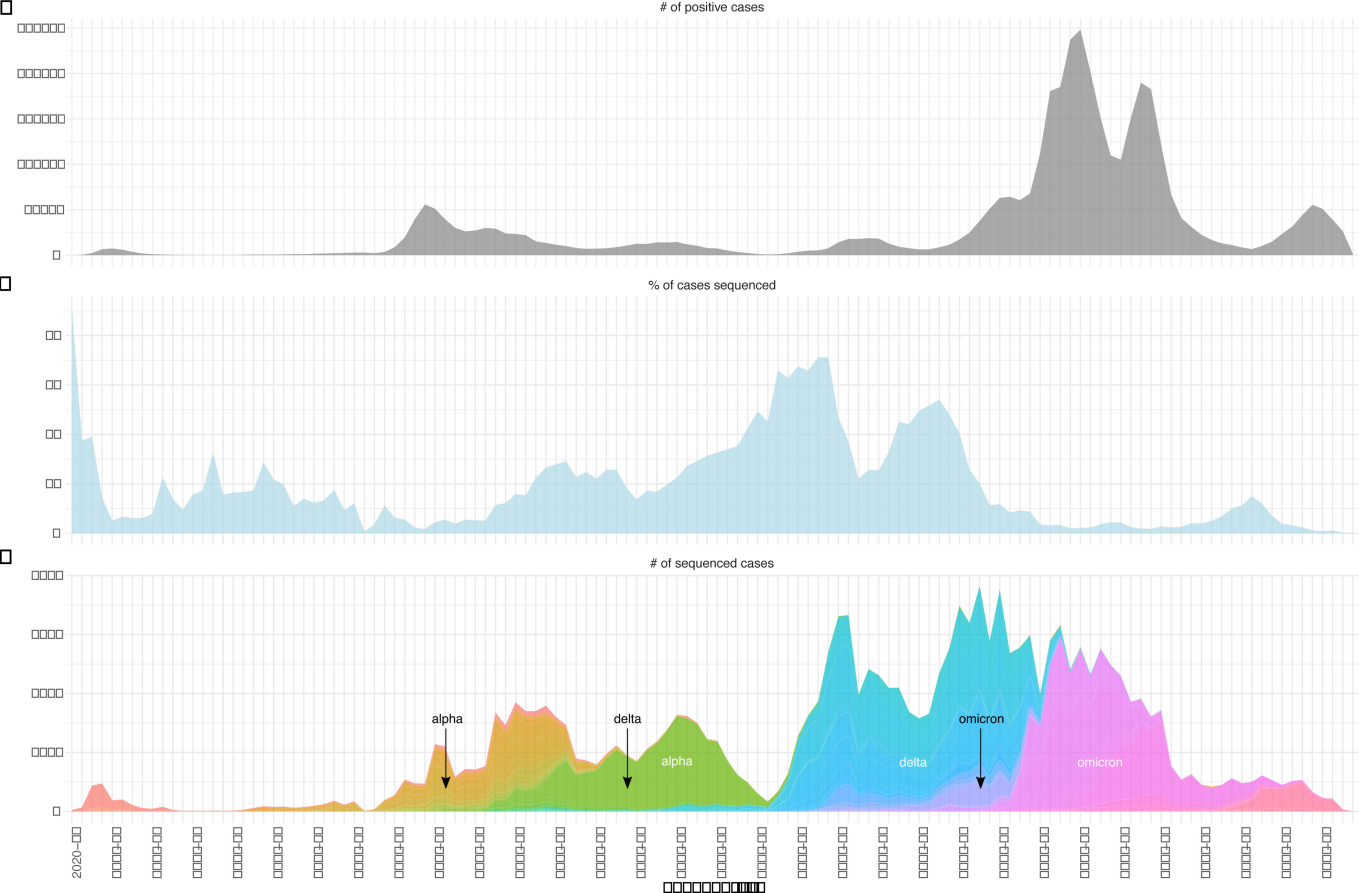
(**A**) Number of positive COVID-19 cases in Switzerland per week. (**B**) Percentage of cases that were sequenced per week. (**C**) Number of sequenced cases per week colored by their Pango lineage assignment. This represents the total number of sequences submitted to SPSP (*n* = 143,260). The black arrows denote the first detection of the VOCs Alpha, Delta, and Omicron. The green wave corresponds to the Alpha wave, the blue and purple colors to the Delta wave, and the pink colors to Omicron.

A total of 143,260 sequences were available for a population of 8.7 million inhabitants over the observed period in our data set (cf. Materials and Methods). The median percentage of cases that were sequenced each week was 7% (Fig. 1). In total, this constitutes 3.6% of all cases (*n* = 3.95 million) for the whole study period.

The first year of the pandemic was characterized by the emergence and persistence of multiple different viral lineages that led to an increase in nucleotide diversity and the lineage diversity index (LDI, as measured by Shannon diversity index, cf. Materials and Methods) (Fig. 1C; Fig. S2 to S6). This diversity on the nucleotide and lineage level was reduced with the first VOC, Alpha, rising to dominance (Fig. S3). All subsequent waves were dominated by a single VOC, with a slow rise in genetic diversity over time and a peak when a new, usually more divergent VOC was introduced (Fig. S3A). In contrast to Alpha, the Pango nomenclature accommodated the diversification of Delta and Omicron by introducing many more sublineages. This leads to a high LDI when all Pango-designated sublineages are considered, but an overall low LDI when these sublineages are consolidated to VOC labels (Fig. S3B). Moreover, as indicated by the arrows in Fig. 1C, Alpha and Delta were already present in Switzerland several weeks before they increased in frequency and truly emerged as the dominant variant. For Omicron, however, this initiation period was much shorter. The pandemic context during the emergence of each VOC was also very different. For example, case numbers were high at the time of introduction of Alpha and Omicron, but low for Delta (Fig. S4). Delta’s rapid growth phase started briefly after a period of decreasing Alpha cases, which coincided with relaxed health and safety measures in Switzerland. This—in addition to its intrinsically higher transmissibility (15)—likely played a role in its rapid increase at the end of June 2021 (calendar weeks 2021-25 onward in Fig. 1C).

### Effect of downsampling on VOC lineage diversity index

To investigate how the observed LDI would have changed with lower sequencing intensity, we downsampled our data set (cf. Materials and Methods).

Naturally, downsampling had an influence on the absolute number of lineages detected (Fig. S6). However, downsampling had only a minimal effect on the LDI during VOC-dominated periods, especially for Alpha and Delta (Fig. S5). The impact of downsampling was stronger during the initial period of the pandemic when the number of available sequences was low. There was also a stronger effect during the later Omicron wave (i.e., once Delta had stopped circulating widely in Switzerland) when downsampling to less than a third of the original effort (less than 50k sequences).

### Effect of downsampling on first detection of VOCs and speed of introduction

**Fig 2:**
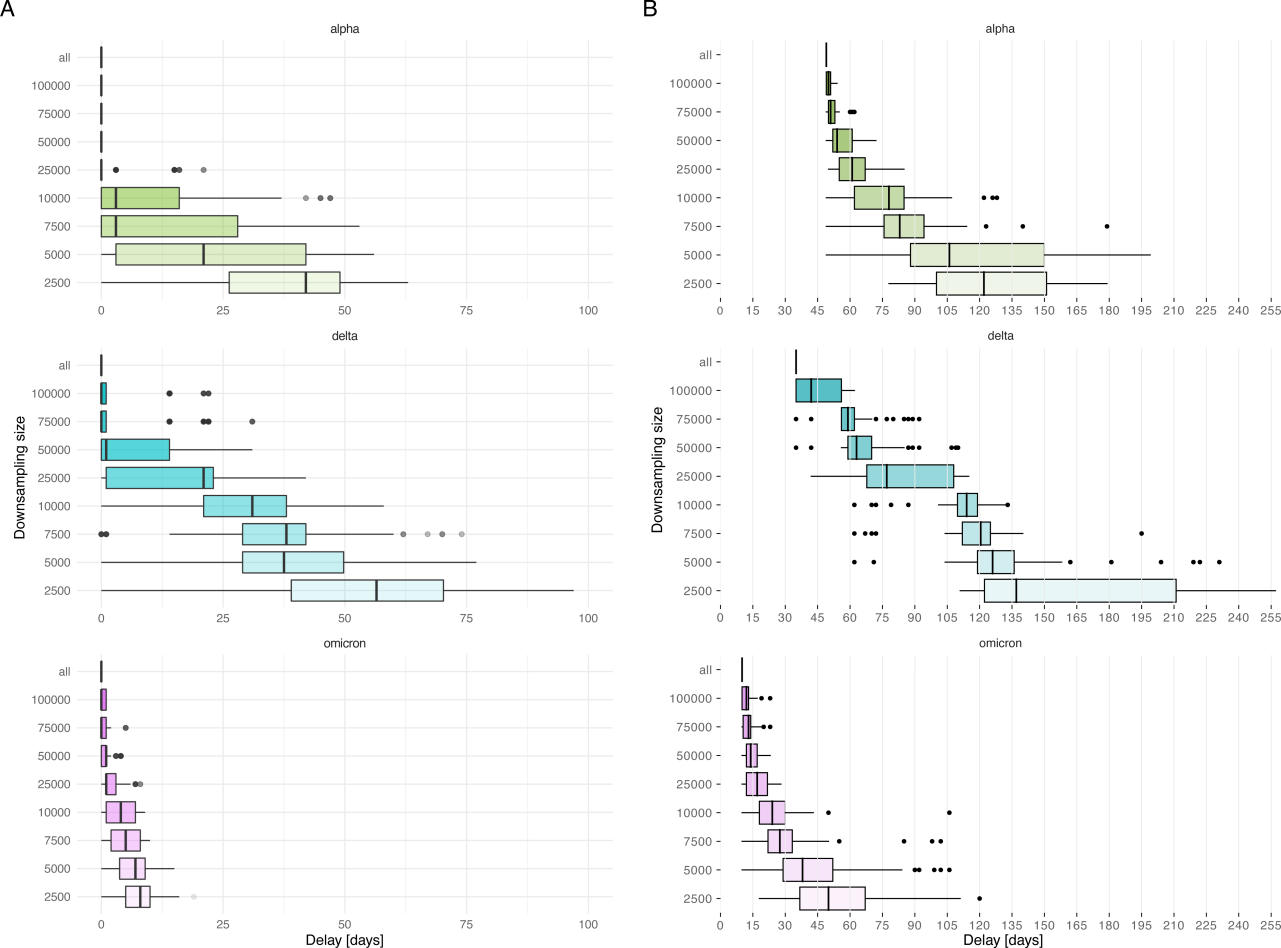
(**A**) Delay in detecting the first sequence of the VOC upon downsampling, as compared to using the full data set (“all”). Delays are shown for 100 iterations at each downsampling size. (**B**) Delay for detecting the first cluster of a VOC after detecting its first introduction, shown for the full data set (“all”) and upon downsampling. Delays are shown for 100 iterations at each downsampling size. Boxplots display the median and interquartile range, with minimum and maximum values shown with whiskers. Color code: delays for Alpha in green, Delta in blue, and Omicron in pink.

We investigated how the first detection of the VOCs would have changed with a reduced sequencing effort, simulated by downsampling our data set. As expected, the delay of detection increased when fewer sequences were available (Fig. 2A). Surprisingly, downsampling to around a third of the available sequences (50k sequences) only led to a 1-day median delay for Delta and Omicron and no delay for Alpha. In general, we note that Delta was more sensitive to downsampling than Alpha and Omicron, as indicated by the longer tail of the delays upon repeated downsampling.

**Fig 3:**
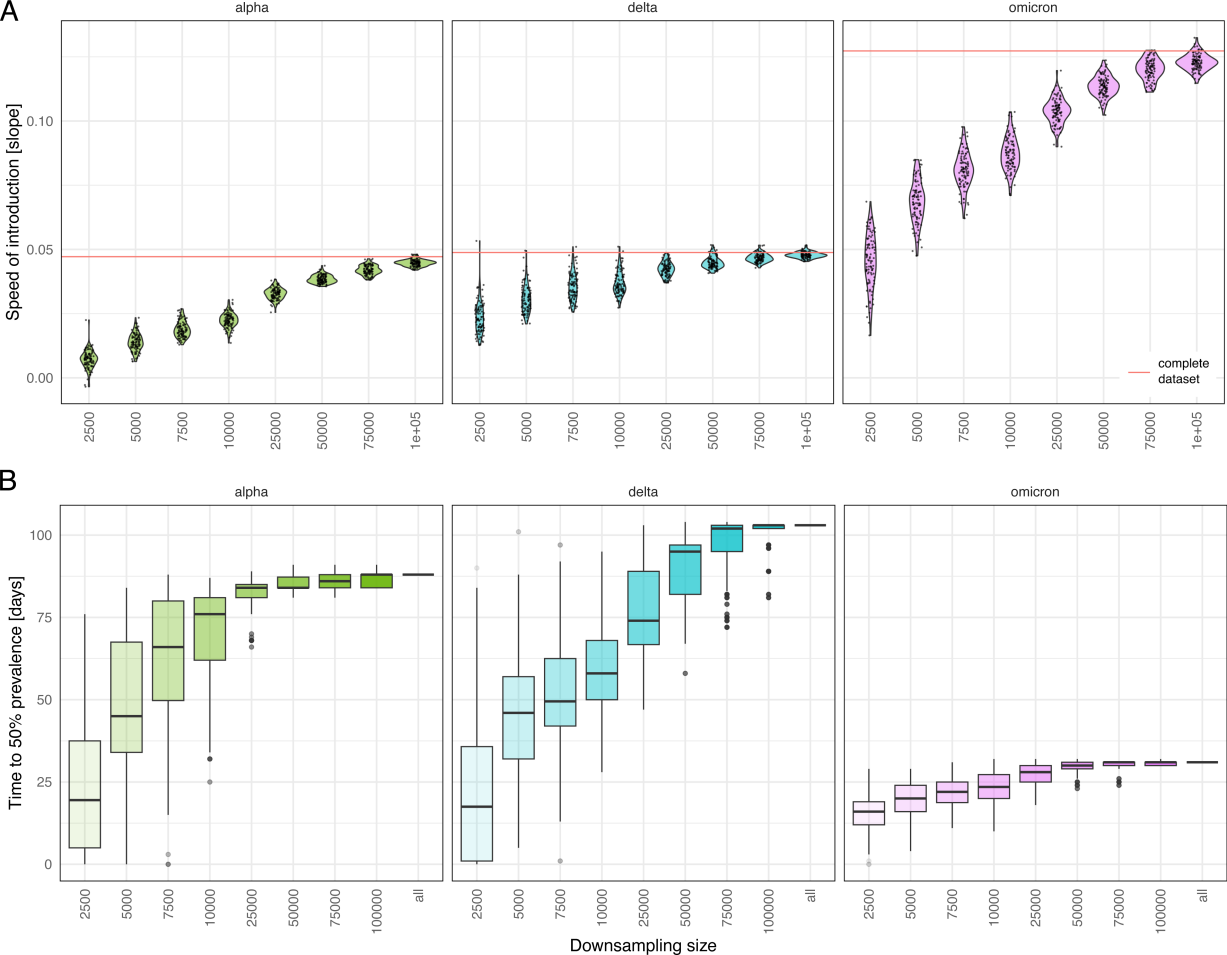
(**A**) The speed of introduction (i.e., the slope of the linear model of the growth phase of each VOC) upon downsampling in relation to the complete data set (indicated by the red line). (**B**) Number of days required to reach 50% prevalence upon downsampling compared to using the full data set (“all”).

We reasoned that differences in the impact of downsampling might be due to differences in how steeply each VOC rises shortly after introduction. Thus, we further investigated the speed of introduction of each VOC, defined as how quickly a newly introduced VOC was established and grew in frequency. While the speed of introduction was similar for Alpha and Delta, it was much faster for Omicron (Fig. 3A). We observed that the speed of introduction also depended strongly on downsampling size, used as a proxy for sequencing effort. A higher sequencing effort was most accurate in capturing the respective growth of the emerging VOCs. Interestingly, the estimates of the downsampled data sets converged with increased sampling size toward the value calculated for the complete data set (red line in Fig. 3), suggesting that the actual sequencing effort captured true VOC dynamics accurately.

We then examined how long it takes for a VOC to reach dominance or 50% prevalence (Fig. 3B). In the full data set, Alpha and Delta required 88 and 103 days, respectively, while Omicron required only 31 days. With increased downsampling, the time needed to reach 50% prevalence decreased. In other words, lineages were seen as dominant earlier when fewer sequences were available. Delta was very sensitive for this measure as the spread of data and also the median time in reaching prevalence strongly increased with each downsampling. However, downsampling to around a third (50k sequences) accelerated prevalence only by 8 days median (4 days for Alpha and 1 day for Omicron), recapitulating the original data still well.

To summarize, we observed that the impact of downsampling on surveillance outcomes was very VOC-dependent (Fig. S7). Downsampling affected Alpha least with regard to the delay in detection and speed of introduction, whereas Delta was affected for both measures. For Omicron, the effect of downsampling on its detection was insignificant, while it was very strong for observing its growth.

These observations mean that differences in the impact of downsampling were not due to differences in growth during the introductory period, as Alpha and Delta displayed a similar speed of introduction for the complete data set but were differently impacted by downsampling. We could observe, however, that Delta had a unique feature. Its introductory period covered the phase in mid-June 2021 (around calendar week 2021-24) when cases (and sequencing) were very low (Fig. 1) and health and safety measures relaxed, giving Delta an additional advantage and leading to its rapid rise. This “gap” might be the reason why different surveillance measures were so strongly affected by downsampling for Delta.

### Effect of downsampling on VOC cluster detection

To understand how the transmission information changes when having fewer sequences, we analyzed the effect of downsampling on the detection of clusters.

We first looked at the delay between the detection of the first cluster of each VOC and the first sequence of that VOC. A cluster is defined as a transmission event between three or more people (cf. Materials and Methods). When considering all available sequences, the delay in cluster detection was very VOC-dependent: 49 days for Alpha, 35 days for Delta, and 10 days for Omicron (Fig. 2B). Figure 2B shows the effect of downsampling on the delay to detect clusters. A commonly asked question during the pandemic was whether new cases were due to endemic transmission or new introductions. This is of epidemiological interest as it can affect the backward and forward tracing strategy. Delay upon downsampling was again more pronounced for Delta. When downsampling to 50k sequences, the delay in the detection of the first cluster was much shorter for Alpha (54 days) and Omicron (14 days) but more accentuated for Delta (63 days). The first Delta clusters appeared in May 2021, but it was not until July 2021 that the VOC gained importance, making it difficult to detect those early clusters with a modest sequencing strategy.

**Fig 4:**
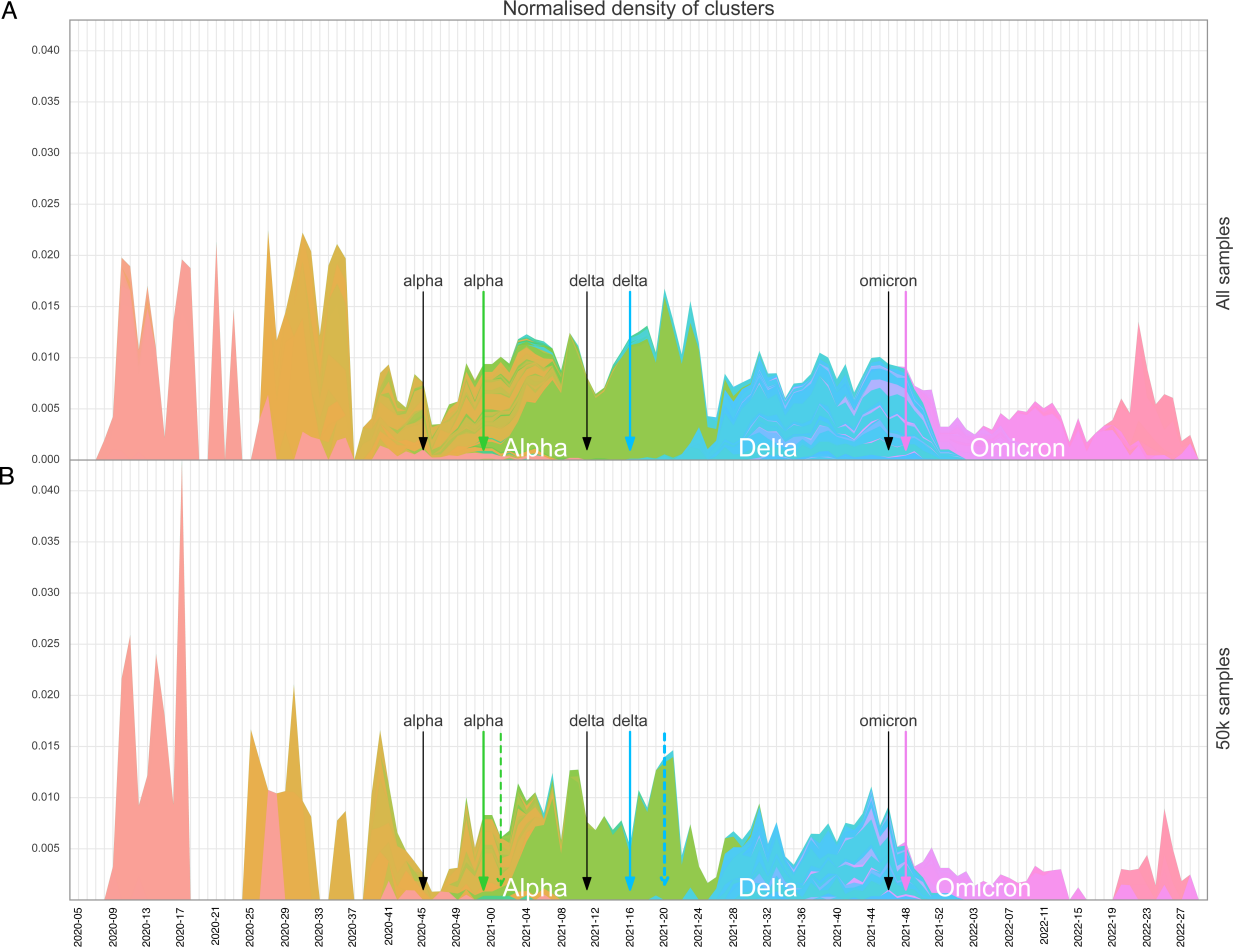
(**A**) Normalized density of clusters per day for all available sequences. (**B**) Normalized density of clusters per day for a sample of 50k sequences. The absolute number of active clusters detected each day has been normalized by the number of sequences obtained in a time window of ±15 days. The colors indicate the VOC (Alpha in green, Delta in blue, and Omicron in pink). Black arrows indicate the date at which the first sequence of each variant was isolated; colored arrows highlight the date at which the first cluster of each VOC was detected when considering all 143k samples; colored dashed arrows show the date at which the first cluster of each VOC would be detected if considering only 50k samples. Note: for Omicron and this particular sample of 50k sequences, the date of the first cluster detection was identical as for the complete data set.

The distribution of the normalized density of clusters is shown in Fig. 4 for the whole data set. Upon downsampling to 50k sequences, we observed a similar pattern of peaks and valleys as with the complete data set, with some additional time windows now being without any clusters (e.g., beginning of July 2020 with two additional weeks without active clusters and in May 2022 with three additional weeks without active clusters) (Fig. 4).

### Effect of downsampling on VOC geographic spread and cluster duration

Cluster characteristics such as the cluster duration and geographic spread help public health organizations understand the spread of an outbreak, e.g., a superspreading event, and thereby argue on the introduction of countermeasures. Thus, it is important to study how these cluster characteristics change when reducing the sequencing effort. For all the available genomes, a total of 3,014 clusters were found of which 54 were long-distance virus movements (LDVM) (1.8%), defined as events that spread a distance >200 km. The median cluster size included three cases (interquartile range (IQR) = 2), while for LDVM events, the median included five cases (IQR = 4.75). On the other hand, for a downsampling size of 50k sequences, 738 clusters were found, from which 17 were LDVM (2.3%). The median number of cases per cluster was three cases (IQR = 1), while it was four cases (IQR = 2) for LDVM events.

Downsampling had little effect on the duration of clusters, a result consistent for all VOCs. The median duration ranged from 10 days for the complete data set to 14 days for 2,500 sequences (Fig. S8).

**Fig 5:**
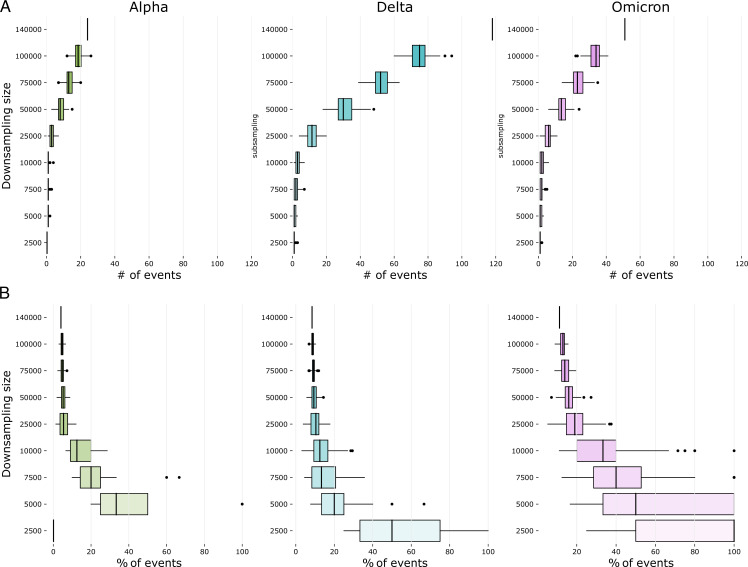
Effect of downsampling on LDVM. (**A**) Absolute number of LDVM detected at each downsampling size. (**B**) Percentage of clusters that were found to be LDVM at each downsampling size.

Downsampling resulted in a reduction of the maximum distance within cases of a cluster (Fig. S9). Most clusters were found to be localized in a single canton. Clusters spreading up to five cantons were less frequent, and clusters appearing in six or more cantons were sporadic. A small yet significant correlation was found between the number of samples in a cluster and the number of affected cantons (Fig. S10). While the absolute number of captured LDVM decreased upon downsampling, their proportion increased, meaning that LDVM got enriched upon downsampling (Fig. 5).

## DISCUSSION

Within a surveillance program, sampling strategy and sample selection are important factors for pathogen sequencing as they influence the ability to detect emerging lineages (14) as well as epidemiological parameters and phylodynamic inferences (16–18). For surveillance of SARS-CoV-2, the European Centre for Disease Prevention and Control (ECDC) recommends a representative sampling approach across geographic locations and demographics for general surveillance for both situation awareness and rare or novel variant detection. This was the chosen approach for Switzerland’s surveillance program. The recommended minimum prevalence to be aimed for in lineage detection is 2.5% (19). In Switzerland, the surveillance program targeted for much of the study period around 2,000 sequences/week, which was just above the ECDC recommendation (1,522 sequences/week) for very rare lineages (1% prevalence) in periods of high case load (>100,000 cases/week) (19). Another study estimated that 5% of cases needed to be sequenced to detect emerging lineages at 0.1%–1% prevalence (20). This threshold was met for most of the period before and during the official national surveillance program until the beginning of 2022 (on average 9.7%, Fig. 1B). This means that Switzerland forms part of the few 6.8% of countries that have sequenced at least 5% of cases in the first 2 years of the pandemic. Forty-five percent of countries have sequenced less than 0.5% of cases (14), highlighting the privileged situation for sequencing-based surveillance in Switzerland.

Concurring with this, we find that simulated sequencing effort by downsampling to around a third of the actual extent had overall only a marginal effect on four of the six surveillance outcomes studied here, especially the first detection date of VOCs as well as their clusters, although this was slightly different for certain lineages based on their unique characteristics and the epidemiological backgrounds within which they emerged (in particular, Delta). Interestingly, this also holds true for cluster duration (i.e., active transmission chains of highly identical strains). The diversity (LDI) was also still recapitulated well with a third of the original sequencing effort during the VOC-dominated periods. Further reducing the sequencing, however, strongly affected specific periods such as the later Omicron wave in terms of LDI. This might be explained by the fact that Omicron has a highly skewed sublineage distribution with a few widespread sublineages co-circulating alongside an assortment of rare lineages, whereas Delta sublineages are more evenly distributed (Fig. 1), leading to the LDI being more sensitive to the disappearance of rarer sublineages with increased downsampling in Omicron.

However, two other outcomes, namely, the geographic spread of clusters (as measured by LDVM) and estimates of the introduction speed and growth of a VOC, especially for Omicron, were more sensitive, and reduced sequencing came at the cost of sensitivity. The latter is because strongly downsampled data might not behave in a linear fashion anymore (cf. Materials and Methods), leading to arbitrary speed estimation values driven by sampling bias. Rather than being a fault, this highlights that accurately estimating the speed of growth of a VOC becomes difficult with sparse data.

In this study, we conducted a retrospective analysis of all available data, rather than simulating real-time surveillance after the fact. This pandemic was the first application of genomic surveillance on such a scale, necessitating a delay in political decision-making and the establishment of surveillance infrastructure. This setup period introduced a discrepancy between the actual collection and submission dates of data due to the retrospective submission of data from this period. Consequently, our analysis specifically focused on evaluating the potential reduction in sequencing efforts under the assumption of real-time data availability, to avoid the confounding effects of the delayed setup period on real-time simulation outcomes.

For this reason, a critical factor for timely lineage detection not assessed in our study is turnaround time (TAT, i.e., the time lag between sample collection and submission of the genome sequence to a surveillance database such as SPSP or GISAID) (21). The median TAT in Switzerland was 18 days (with IQR of 14 days) over the period of the official national surveillance program. Assuming a TAT of 21 days, the probability of lineage detection before it reaches 100 cases was estimated as 0.51 and 0.96 if 1% or 5% of cases were sequenced, respectively, as simulated with data from Denmark (14). This highlights that other important factors than just the amount of sequencing should also be targets of surveillance optimization, which are harder to assess and influence in non-centralized countries such as Switzerland where sequencing was performed by many different public and private laboratories.

We showed that the effect of sequencing effort differs for different surveillance outcomes but that many outcomes could have been recapitulated with a reduced sequencing effort assuming real-time data availability. Such a study was possible due to Switzerland’s high per capita sequencing effort. However, we believe that the results are still transferrable to other European countries as the overall viral dynamics were similar across Europe with overall similar circulating lineages. Indeed, we believe our results are encouraging for countries with less resources than Switzerland. A national surveillance program needs to strike a balance between societal benefits and program costs, and as we have shown, some outcomes require more sequencing effort than others. To achieve a cost-effective program, the desired outcomes of surveillance should be clearly defined and sequencing targets set accordingly. The SARS-CoV-2 pandemic was unprecedented, necessitating swift adaptation and learning amid the crisis, inevitably leading to mistakes. However, it also served as a vital learning opportunity, ensuring that for any future epidemic or pandemic, we are now better equipped to establish surveillance systems in a more cost-effective and efficient manner.

We were able to conduct this study thanks to the coordinated SARS-CoV-2 surveillance program led by the National Reference Lab for Emerging Viral Infections and the centralized data collection approach via the Swiss Pathogen Surveillance Platform. Such a platform served to coordinate the data sharing following FAIR principles (findable, accessible, interoperable, and re-usable) (12, 13), reduced burden on laboratories with a single point of entry prior to data re-sharing to international archives, and efficiently liaised with public health authorities. A centralized infrastructure for data collection and processing of genome and epidemiological data is of crucial importance during a public health crisis.

## MATERIALS AND METHODS

### Data

All sequences (consensus sequences) shared with SPSP (https://www.spsp.ch) until the 5th of August 2022 were used (*n* = 143,260) in this study. Genomes sequenced with the explicit purpose of outbreak investigation were removed. The earliest genome collection date was 24 February 2020, the latest 1 August 2022. Genomes with incorrect collection dates (obvious data entry errors) were excluded. Quality control was done by the submitting laboratories (only genomes passing quality control of the individual labs are intended to be shared with SPSP). Pango lineage assignments were performed on SPSP in regular intervals and correspond in the data set to the latest Pangolin version available on 5 August 2022 (22). Additionally, Swiss COVID-19 case data were taken from the FOPH dashboard (https://www.covid19.admin.ch/, last accessed 20 September 2022).

### Downsampling and key measures

The complete data set was downsampled to sizes ranging from 2,500 to 100,000 using random uniform sampling and 100 iterations. VOC labels (Alpha, Delta, and Omicron) were given to all genomes with assigned Pango sublineages associated with a given VOC. The introduction date was determined as the earliest collection date of a given VOC in the data set. The speed of introduction was calculated as the slope of a linear regression model of the log of exponential growth phase of a given VOC. The end of the exponential growth phase was determined based on the complete data set as 2021-03-01 (week 2021-09) for Alpha, 2021-08-01 (week 2021-30) for Delta, and 2022-01-01 (week 2022-01) for Omicron.

The lineage diversity was calculated as the Shannon diversity index (23) using the R package vegan (24). It accounts for both lineage richness and evenness, i.e., the number of lineages as well as their proportion. For example, the diversity in two samples with the same number of lineages would be greater in the sample with even proportions for each lineage compared to the sample with a few dominant and many rare lineages.

The nucleotide diversity was calculated for whole genome alignments [produced with mafft v7.505 (25) of all sequences per calendar week using the R package pegas (26).

### Cluster definition

A three-step method was used to identify sequence clusters. First, sequences with 0 single-nucleotide polymorphism (SNP) distance (including missing regions) that were collected maximally 20 days apart were grouped to obtain mutually exclusive clusters. These early clusters should have a minimum of three sequences. At this point, if a SNP difference occurred in a region that was missing in an otherwise identical sequence, this was counted as a SNP difference of 1, and the two sequences would be placed in different clusters. To overcome this and account for the possible mutations within a transmission chain, 0-SNP-clusters were expanded by adding sequences at 1 SNP distance. This resulted in multiple non-mutually exclusive clusters with overlapping sequences. Finally, the expanded clusters sharing sequences were merged using hierarchical clustering to create mutually exclusive superclusters. To ensure the quality of the superclusters, superclusters containing sequences collected more than 30 days apart were removed from the study. This time window was selected based on the estimated mutation rate of SARS-CoV-2 of 1.1  ×  10^−3^ subs/site/year (i.e., around two to three mutations per month) (27). To maximize the number of superclusters, the duration of 0-SNP-clusters was studied by comparing the number of sequences, cluster counts, and percentage of superclusters spanning more than 30 days for different duration thresholds (Fig. S11). A threshold of 20 days for the 0-SNP-clusters was found to maximize the number of sequences analyzed and the number of clusters found and to minimize the resulting number of superclusters exceeding 30 days.

### VOC cluster detection dates and geographic spread

The delay in VOC cluster detection was computed in relation to the first detection of a VOC sequence. The effect of downsampling on the detection of the first VOC clusters was analyzed by finding the distribution of the first cluster detection dates in the downsampled data sets. The downsample size of 50k samples was considered the smallest size in which the VOC detection dates could be retrieved without major delays (i.e., 1-day median delay for Delta and Omicron and no delay for Alpha; cf. Results on the first detection of VOCs upon downsampling and Fig. 2A). Therefore, this size was selected as the downsample size for further analysis.

Clusters containing the same sequences in the complete data set and a downsampled data set (50k sequences) were matched. Discrepancies in cluster matching can arise because (i) large clusters lasting longer than our 30-day threshold are discarded but are found in part in the downsampled data set or (ii) larger clusters from the complete data set were split into several clusters of shorter duration in the downsampled data set given that the common sequences are not in the sample anymore.

The distance between the cantons affected by clusters was computed as the Euclidean distance in kilometers between the capital of each canton.

### Normalized density of clusters per day

Clusters were determined for the complete data set as well as the 50k downsampled data set (see above). A cluster was considered active during the period between the isolation of its first and last sequences. Given that the sequencing effort was not consistent during the study period, only considering the absolute counts of clusters per day would introduce a bias toward the periods with more sequencing effort. Therefore, the absolute number of clusters active each day was normalized by the number of sequences isolated in a time window of ±15 days (30 days being the estimated maximum duration of a cluster).

## SUPPLEMENTAL MATERIAL

Supplemental material spectrum.03628-23-s0001.pdf Figures S1 to S11.

ASM does not own the copyrights to Supplemental Material that may be linked to, or accessed through, an article. The authors have granted ASM a non-exclusive, world-wide license to publish the Supplemental Material files. Please contact the corresponding author directly for reuse.
